# Methodology for Revealing the Phases and Microstructural Constituents of the CMSX-4 Nickel-Based Superalloy Implicating Their Computer-Aided Detection for Image Analysis

**DOI:** 10.3390/ma13020341

**Published:** 2020-01-11

**Authors:** Agnieszka Szczotok, Hannah Reichel

**Affiliations:** 1 Faculty of Materials Engineering, The Silesian University of Technology, Krasinskiego Str. 8, 40-019 Katowice, Poland; 2 Fakultät Ingenieurwissenschaften und Informatik, Osnabrück University of Applied Sciences (Hochschule Osnabrück), Albrechtstraße 30, 49076 Osnabrück, Germany; hannahreichel18@gmail.com

**Keywords:** superalloy, CMSX-4, phase revealing, etching, metallography

## Abstract

The paper presents the findings of a research on the selection of methodology for revealing the microstructure in metallographic investigations on the example of the single-crystalline CMSX-4 nickel-based superalloy. A set of chemical and electrochemical methods of etching has been selected. The metallographic specimens from the analyzed material have been treated with the etchants. After every etching procedure, microphotographs of the microstructure were taken by means of an optical microscope and a scanning electron microscope. Both useful and disadvantageous effects of etching with the respective etchants have been displayed. The etchant application for a qualitative and quantitative analysis has been considered on the basis of the enclosed microphotographs. As a result, examples of a computer-aided detection of the phases and microstructural constituents present in the analyzed CMSX-4 alloy for the selected revealing methodologies have been demonstrated. The described investigations enable a better understanding of the essence of the selection of the microstructure revealing methodology and its influence on the obtained results.

## 1. Introduction

Metallographic analysis can be used as a tool to help identify a metal or alloy, to determine whether an alloy was processed correctly, to examine multiple phases within a material, to locate and characterize imperfections such as voids or impurities, and as a result, to determine the product’s reliability (Quality Control) or to observe damaged or degraded areas in failure analysis investigations in order to establish why a material failed (Metallographic Failure Analysis) [1]. The appropriate preparation and analysis of the microstructure is a critical component of many material characterization efforts, whether the ultimate goal is to develop new materials, to advance our understanding of an existing material or to determine why a failure has occurred. More than any other attribute, the imaging of the topographical or microstructural features as well as the structural studies by means of metallography are indicative of the properties and performance of the examined material. Conversely, insufficient attention to microstructure control can lead to unpredictable properties, inconsistent behaviour and material failure [2]. Consequently, the task of metallography is to ascertain the structure of the material by means of macro and microscopic procedures. That is why both optical and scanning electron microscopy with energy-dispersive X-ray analysis (SEM/EDX) can be useful in metallographic analyses. In order to be able to examine a material, achieve an accurate analysis and come to satisfactory conclusions about its quality, obtaining ‘the true microstructure’, meaning an undisturbed material surface, is an important initial step in metallography [3]. The preparation goals should include the following: all structural elements must be retained, the surface must be without scratches or deformation, no foreign matter may be introduced on the specimen’s surface, and the specimen must be plane and highly reflective.

It is well known that the microstructure of a material depends on the type of the material and its manufacturing processes. In the case of γ’- strengthened nickel-based superalloys, the size and volume fraction of the γ’ phase are critical input parameters for models of the mechanical properties of Ni-based superalloys [4]. That is why metallographic examination is a necessity for the understanding of the properties and their enhancement. Metallographic etching is a technique used to highlight the features of metals at microscopic levels. To reveal a specific microstructural feature, like the γ matrix or γ’ phases, numerous etching methods have been developed, among which chemical etching is certainly the easiest and the most widely used. This technique utilizes a controlled corrosion process driven by the electrochemical potential differences between surface areas with chemical or physical heterogeneities. Etching induces selective dissolution or preferential staining of the present phases [5]. The chemical or electrolytic action of an appropriate reagent (etching) is usually necessary to reveal the microstructural features of superalloys. Sample preparation requires a certain degree of skill and experience, due to the high chemical resistance of most superalloys. In the case of electron microscopy, since the differences in composition between the γ matrix and γ’ phases are relatively small, the contrast in the absorbed or backscattered electron images is only slight, and it is often necessary to lightly etch the specimens before examination [6]. However, the polished specimen should be first examined unetched. 

According to [7], not just the average size, but the size distributions of γ’ phase as well, play an important role in the mechanical properties and the microstructural evolution. It is therefore of substantial importance to be able to measure the particle size distribution. An accurate and efficient quantitative analysis of the microstructure in superalloys by means of image analysis is a challenge. The processing and image analysis techniques for industrial and scientific environments have evolved rapidly [8]. The course of investigations is usually as follows: (i) firstly, image formation performed by means of microscopy methods (i.e., scanning electron microscopy, optical microscopy); (ii) secondly, the image is preprocessed by different filters and transformations; (iii) thirdly, segmentation is necessary to obtain the extraction of the feature of interest; and (iv) finally, classification used to quantify the microstructural feature and interpretation. 

Most existing techniques for characterizing particle size distributions from images require tracing of the image features to extract quantitative information. The separation of features in an image from the background is known as ‘segmentation’. Segmentation of intensity images is accompanied by a reduction of information, consisting in classifying the pixels as either belonging to a feature of interest (e.g., the γ’) or to the background. In materials sciences, image segmentation is often performed by hand, which usually makes it the most tedious and expensive part of quantitative characterization because it is time and labor consuming [9], especially for larger numbers of particles of γ’ phase. Segmentation is indirectly related to the effects of the applied etching for the revealing of the microstructure. Unfortunatelly, there are difficulties in the ascertaining of the γ/γ’ microstructure. The first problem are intensity changes within the image, as the etchant used to highlight the γ or γ’ phase can etch the material at different rates within the metallographic sample, varying the contrast in intensity for the γ and γ’. 

A considerable amount of studies has been published on the microstructure of Ni-based superalloys, e.g., [10,11,12,13,14], but one can very rarely find information about the limitations of the presented work, the sources for error or an explanation why the authors have chosen those specific ways of metallographic analysis. It is rather unusual to find literature data describing the role of the sampling strategy or the sample preparation and etching for the metallographic analysis of Ni-based superalloys. Among those few, there are works [15,16] suggesting that the etching time and the etchant type are two major factors which can remarkably affect not only the morphological observations of the precipitates but also the accuracy of the quantitative analysis. This study seems to be one of the not numerous works which outline the critical role of etching in the revealing of phases and microstructural constituents on the basis of one selected Ni-based superalloy CMSX-4. It provides an opportunity to see and consider the results of 14 etching methods applied on the same material in one spot to compare the differences in the microstructure revealing between them. The purpose of this paper is also to describe a combination of the sample preparation method, the imaging technique and the segmentation algorithm that may be used to characterize the γ’ phase in Ni-based superalloys on the basis of the well known commercial CMSX-4 superalloy.

## 2. Materials and Methods 

**Figure 1:**
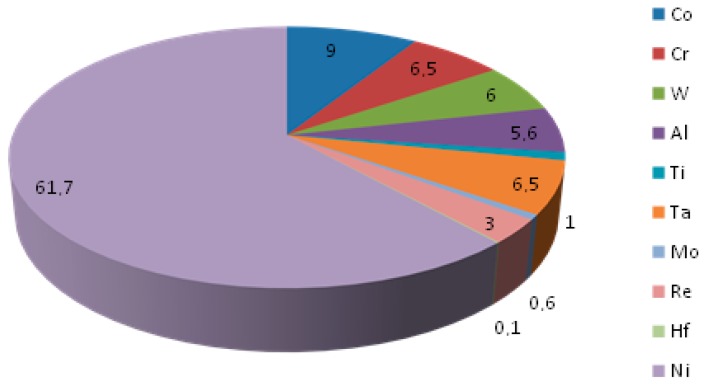
Pie chart representation of CMSX-4 chemical composition (wt %).

Nickel-based superalloys, due to their high heat resistance, strength and creep resistance at high temperatures, as well as toughness and corrosion resistance, are very often used for the construction of gas turbine engines. CMSX-4 is a second generation single-crystal superalloy containing 3 wt pct Re. This alloy is derived from CMSX-2, employing the beneficial strengthening effects of Re. It was established in the early 1990s and is used for single-crystalline turbine blades. Single-crystalline CMSX-4 is typically fabricated via investment casting and has been extensively developed to enhance the high-temperature properties. In addition, the microstructure is optimized by special heat treatments [17]. The single-crystalline CMSX-4 nickel-based superalloy is one of the most popular representatives commonly used for the manufacturing of aircraft engine hot-zone turbine blades developed by the Cannon Muskegon Corporation. The significant world wide application of CMSX-4 is mainly a result of good single crystal castability with moderate to high thermal gradient and production experience similar to that of CMSX-2 and CMSX-3; oxidation resistance (both bare and coated) at least as good as that of the CMSX-2 and CMSX-3 alloys; improved hot corrosion resistance; as well as excellent phasial stability, tolerant of rare earth elemental ppm residual additions for enhanced bare oxidation resistance and thermal barrier coating adherence [18]. Like all materials intended for the use in the aerospace industry, CMSX-4 must be supplied by approved and reliable suppliers. In addition, these materials are subject of very stringent quality control. The superalloy under investigation was available as-cast in the form of a round cylindrical bar with [001] orientation obtained with the use of the Bridgman method. Figure 1 provides the information about the chemical composition of the studied CMSX-4.

The use of qualitative studies of the Ni-based superalloy microstructure is a well-established approach. The two-phase structure of the CMSX-4 single-crystal nickel-based superalloy has been identified by microscopy and X-ray diffraction in many works, e.g., [19,20,21]. The microstructure of the material in its as-cast state is composed of gamma (γ) and gamma prime (γ′) phases as well as γ/γ’ eutectics as microstructural constituents. The γ′ phase (Ni_3_(Al,Ti))—primary strengthening phase—is coherent with the matrix—γ phase. 

The metallographic samples for both ligh microscopy (LM) and scanning electron microscopy (SEM) observation were prepared by way of grinding on abrasive papers and polishing on diamond pastes according to the scheme and parameters presented in Table 1.

Table 2 includes all applied etchants, their composition and etching techniques that the authors have found useful in the examining of the microstructure of the Ni-based alloy. This is not meant to be an exhaustive list, but merely what the authors commonly use for the superalloys. The American Society for Testing and Materials (ASTM) produce industrially recognised standards for the analysis of the material microstructure. The authors have verified the compliance of the proposed etchants (Table 2) comply with the recommendations of the ASTM [22]. The authors declare that to the best of their knowledge, etchant no. 1 is known as the Adlers etchant and no. 2 has no name. Beside those two mentioned ones, the rest of the etchants from Table 2 have their own ASTM numbers [23].

The microstructural observation of the CMSX-4 superalloy in the as-cast state was performed by optical microscopy (Olympus GX71 light microscope, in lower magnification and scanning electron microscopy (FE SEM HITACHI 4200 equipped with an X-ray spectrometer VOYAGER 3500 of NORAN, in higher magnification. 

To compare the effect of the etchant and the etching conditions on the microstructural observations and their usefulness for the possibilities of extraction of the analysed phases and microstructural constituents for further analysis of the selected examples, image processing was performed. The transformations of the digitalized micrographs of the CMSX-4 microstructure for phases and γ/γ’ eutectics binarization were carried out with the use of the Met-Ilo computerized image analyser [24,25] developed at the Silesian University of Technology.

## 3. Results

**Figure 2:**
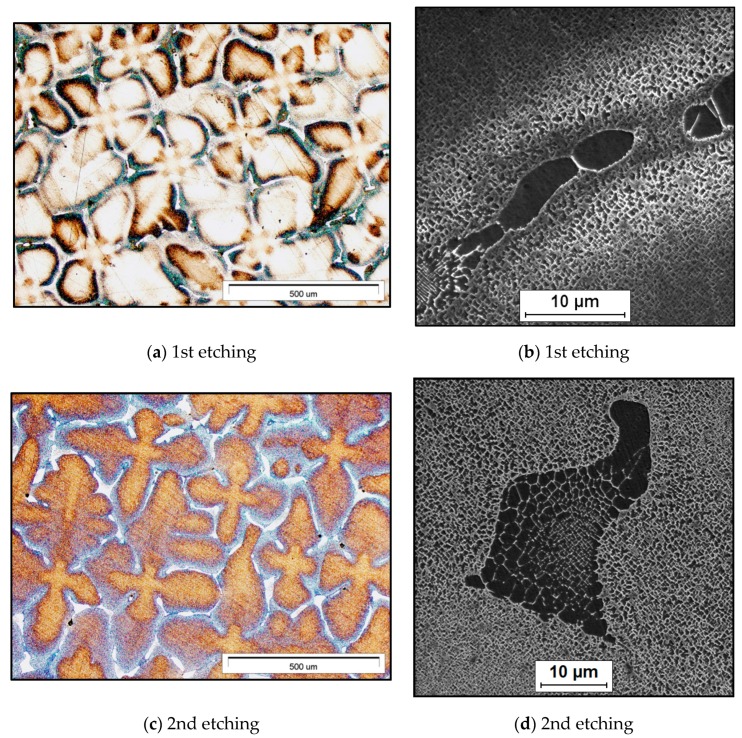

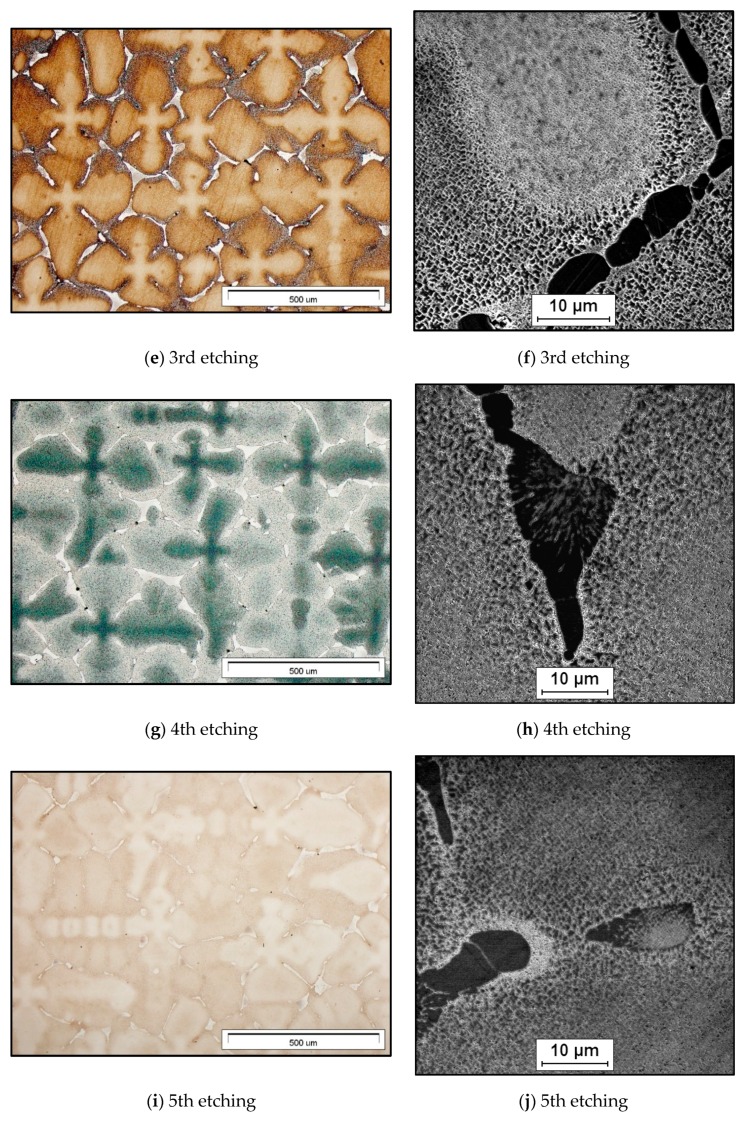

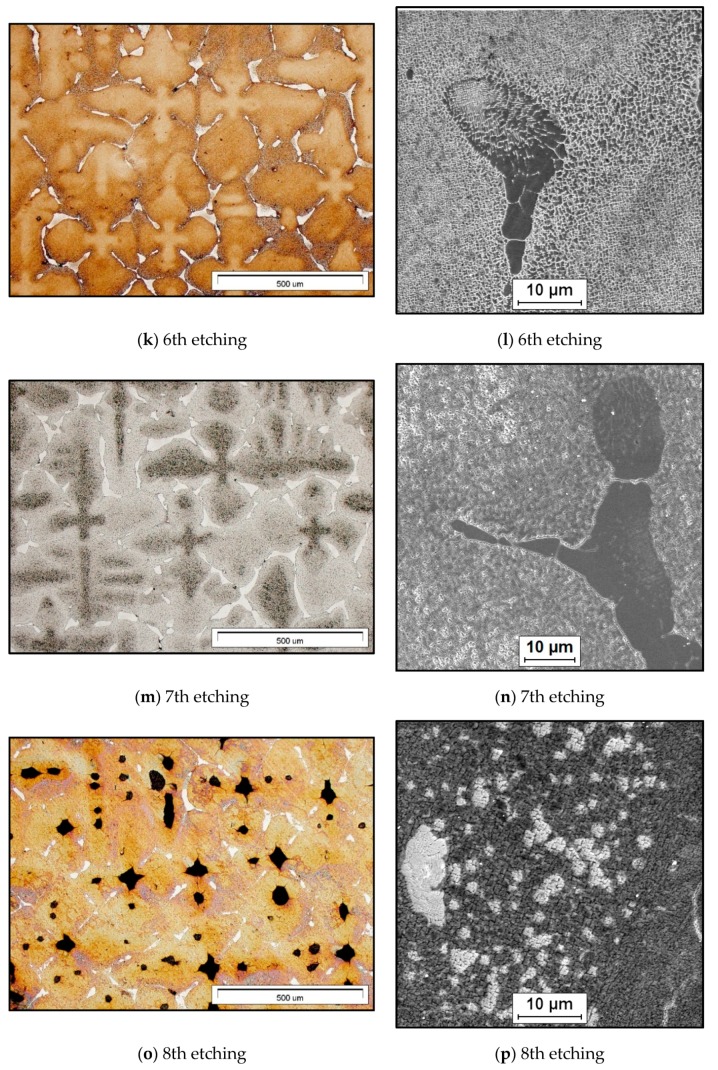

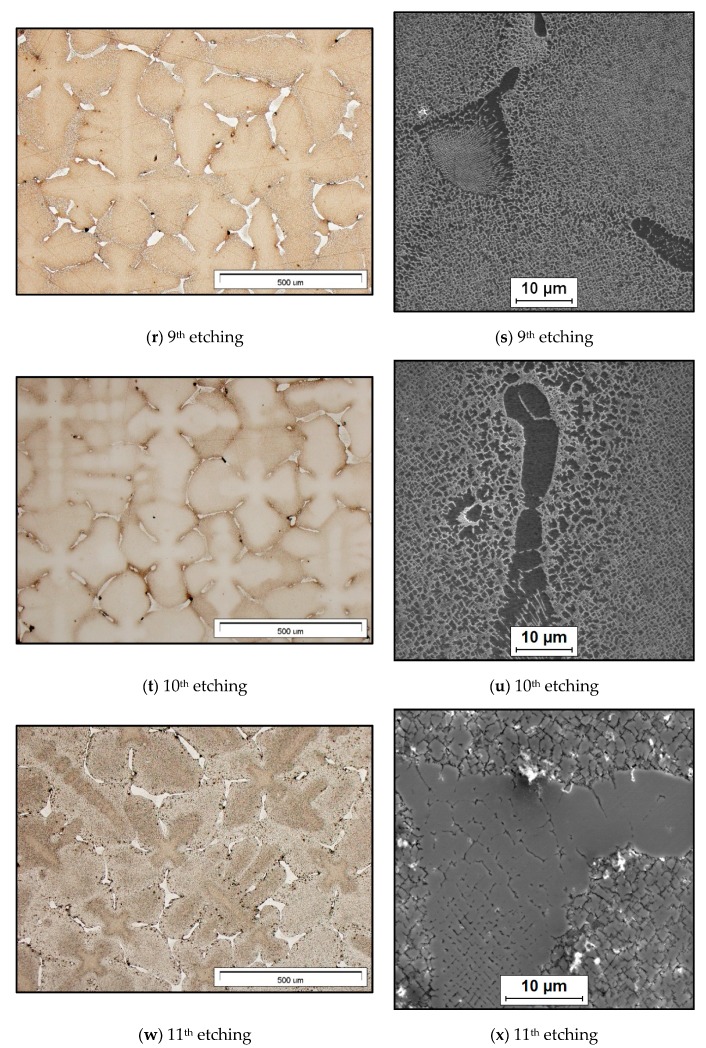

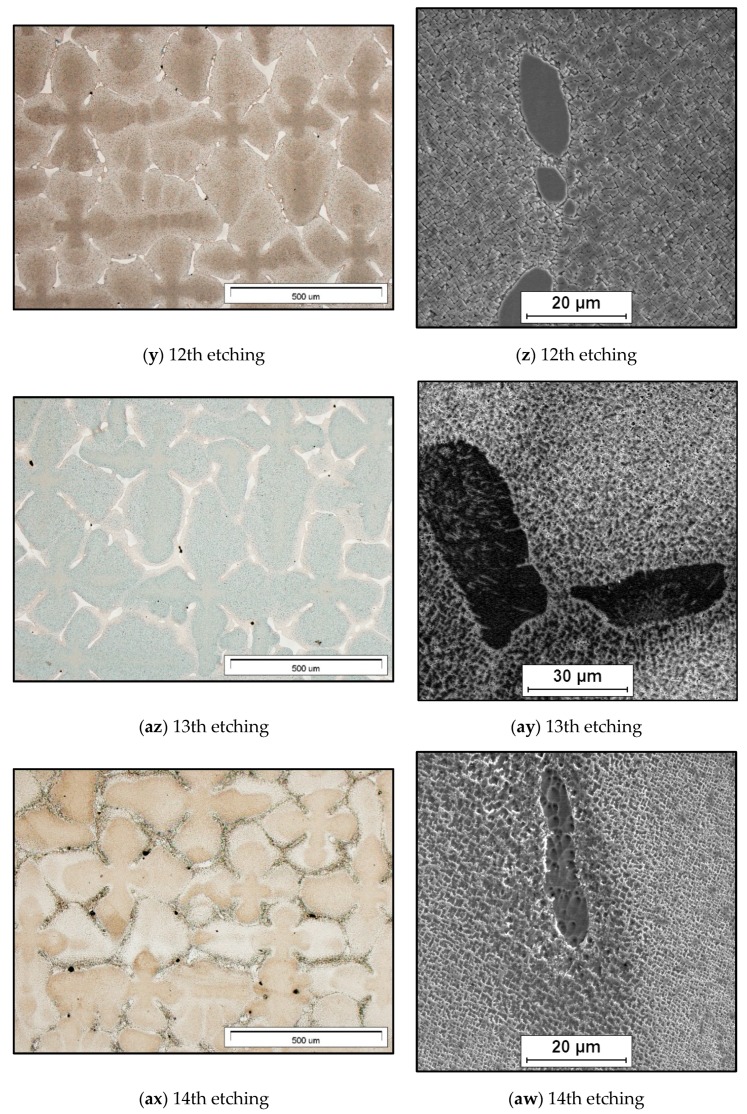
The cross-section microstructure of the as-cast single-crystalline CMSX-4 superalloy: **a**,**c**,**e**,**g**,**i**,**k**,**m**,**o**,**r**,**t**,**w**,**y**,**az**,**ax** (LM, BF); **b**,**d**,**f**,**h**,**j**,**l**,**n**,**p**,**s**,**u**,**x**,**z**,**ay**,**aw** (SEM, SE).

The diversified effects of the application of the various methodology for the revealing of the CMSX-4 microstructure can be seen in the representative microstructures of the investigated alloy (Figure 2). Dendritic segregation formed due to a significant chemical heterogeneity is very often evaluated as typical for cast superalloys. The as-cast microstructure of the investigated superalloy is shown in Figure 2. As expected, the superalloy exhibited high segregation, which was confirmed by a dendritic structure—see Figure 2 and the micrographs from the light microscope (LM). 

The as-cast microstructure of the investigated single-crystal CMSX-4 superalloy in cross section consists of an array of cut dendrite trunks (visible by LM observation) with a fine γ/γ’ structure, interdendritic regions with a coarser γ/γ’ structure, pools of γ/γ’ eutectic and coarse γ’ islands (revealed by SEM observation) (Figure 2). The primary dendrite arms are approximately parallel to the axis of the crystal growth. The γ/γ’ eutectic pools have been noted in the interdendritic areas. The presented micrographs highlight the shape and size of γ’ phase precipitates, which are more regular and smaller in the dendrite cores than in the interdendritic regions, as well as in the γ/γ’ eutectic pools. This morphology of γ’ phase precipitates is due to the growth kinetics in correlation with the segregation level of the alloying elements, while the eutectic γ/γ’ fractions and coarse γ’ islands are developing during the solidification in the interdendritic regions.

Looking at the results of the etching, it can be pointed out that almost all the etchants provide good and very good effects taking into account the revealing of the dendritic structure and the observation in lower magnification, with the exception of etchants no. 7 and 8. The latter seem to interact with the surface of the sample too much and too aggressively. The material was dissolved too fast when immersed in the chemical solution. The time of the etching suggested in the recipe was 10–15 s at 6V using a stainless-steel cathode and a platinum or nichrome connection to the specimen. The authors started with a shorter time, up to only several seconds. Despite of that, the result of etching was unsatisfactory. It might be the effect of a still too long etching time, the freshness of the etchant prepared just before the etching or the thickness of the slice of the material which was etched. The authors decided to present this effect to show that the conditions of the etching have a great impact on the obtained results from revealing the microstructure of the material.

It is possible that the etched surface can be quite sensitive to the relative amounts of the two solutions, which were applied one after the other (reagent no. 13 in Table 2; Figure 2az,ay).

The dendrites and interdendritic areas with γ/γ’ eutectic seem to be better visible with more contrast (Figure 2a,c,e,k,az,ax). In some cases, primary and secondary dendrites are more conspicuous (Figure 2e,k,m,t,w,y,ax). The details, such as the morphology of γ’ phase and γ/γ’ eutectic, might be observed in higher magnification with the use of SEM (Figure 2b,d,f,h,j,l,n,p,s,u,x,z,ay,aw). The presented micrographs from SEM have been recorded in a selected range of magnifications to display the differences in the etching of various microareas, which appear after the application of some reagents (see Figure 2b,u,aw); the interdendritic areas with γ/γ’ eutectic pools are more noticeable than the dendritic areas (Figure 2b,u), or the interdendritic areas with γ/γ’ eutectic pools react with the etchant more than the dendritic areas (Figure 2aw). Some of the applied etchants result in blurring of the microstructure image (Figure 2h,j,ay). Moreover, in the case of etchant no. 11, some unexpected, visible, white, fine lint appears (Figure 2x) not belonging to the microstructure. This could be the effect of inadequate rinsing of the sample after etching or the result of inadequate combining of all ingredients of the etchant and the reaction with the material. Moreover, the exhibited results of etching show that various etchants react with the material by two different ways: Some etchants dissolve the γ matrix (Figure 2h,n,p,x,z,ay) and others dissolve the γ’ phase (Figure 2b,d,f,j,l,s,u,aw). Both kinds of etching are useful for a qualitative description of the microstructure, but as can be found in the literature, the etchants that selectively dissolve the γ’ phase have a better potential for an accurate quantitative analysis of the superalloy microstructure. On the basis of all the 14 performed experiments with a selection of etching methods, it can be figured out that the best results in terms of a good visibility and clarity (no blurriness) of all the phases and microstructural constituents in the whole field of view with the use of SEM have been provided by the application of etchants no. 2, 6 and 9.

The research in the field of computer analysis of microscopic images, in the field of automation of the acquisition process and on-line image analysis as well as application of the gained knowledge in practice is developing. A digital image acquired from a microscope camera is often described as a raw image prior to processing. It is important to focus the optics correctly using LM or to select the appropriate conditions of observation using SEM, in order to capture the fine details in the image. The human eye is used to perform this task. After digital images have been captured and prior to the initiation of the processing algorithm applications, each image should be evaluated with regard to its general characteristics, including noise, blur, background intensity variations, brightness and contrast and the general pixel value distribution (histogram profile).

A digital image of a microstructure is a collection or set of different pixels. We group together the pixels that have similar attributes using image segmentation. In terms of quantitative microstructural characterization, many microscopical techniques suffer from the fact that the resulting images cannot be segmented in an automated manner. The features in properly segmented images may be measured using pixel counting techniques in many image processing software packages. With the proper choice of imaging technique and image segmentation algorithm, it is possible to collect realistic measurements of the particle size distribution in a semi-automatic manner [9]. The key challenge when using automated segmentation algorithms is ensuring the segmentation produced accurately represents the features to be measured. This is widely considered the most difficult task in this type of image analysis; methods capable of segmenting images for one application often do not work in others [26,27]. 

It is also important to note that, for automated image segmentation, the imaging technique used should provide clear and consistent differences between the feature of interest to be analyzed and the background, and for that reason, the applied etching is so essential. 

**Figure 3:**
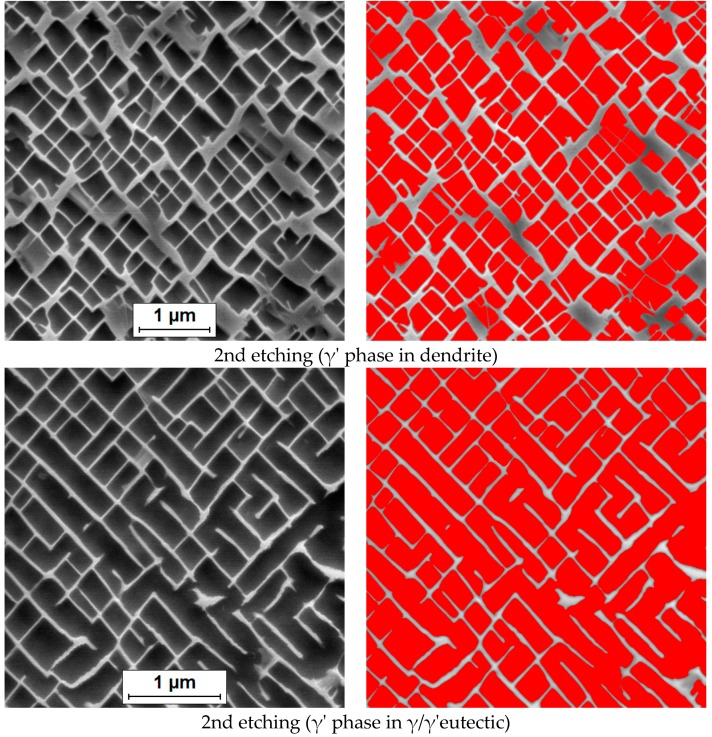

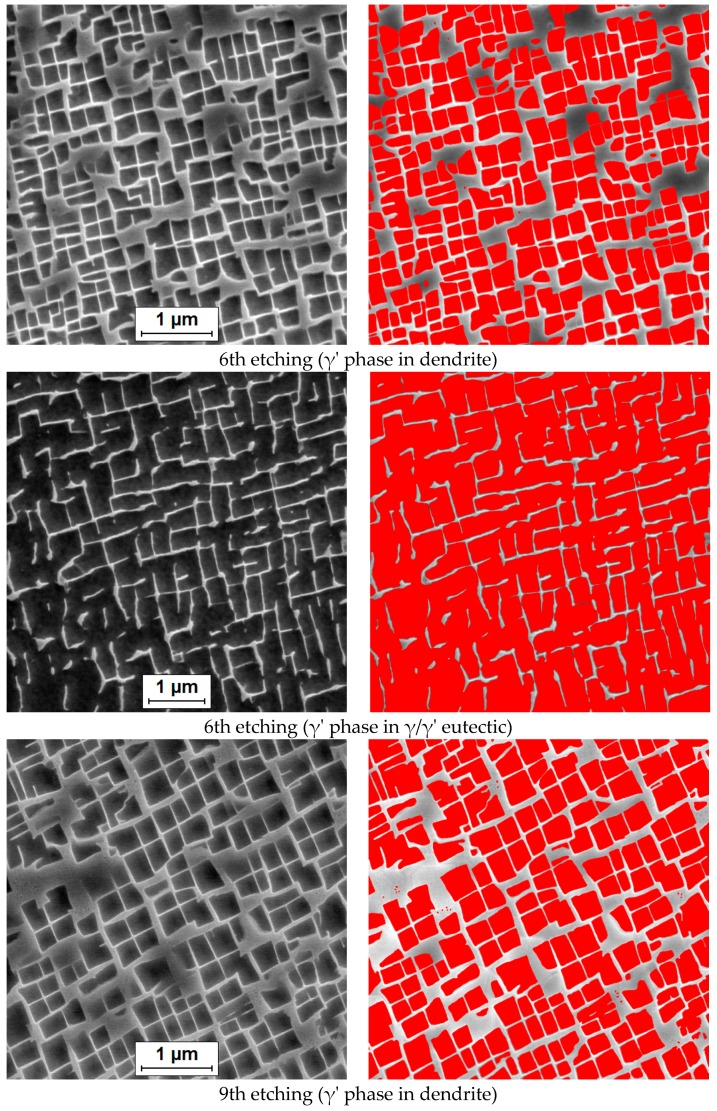

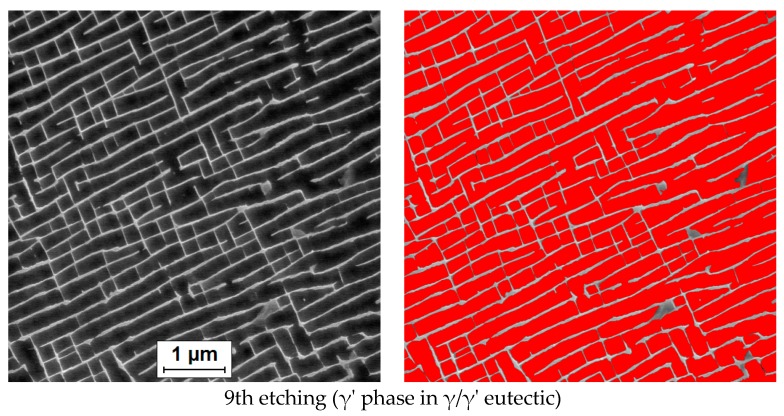
The raw images of the CMSX-4 superalloy and the final images with detected microareas for a quantitative analysis. The microareas of the CMSX-4 selectively etched to remove γ’ phase (left), segmentation of the γ’ phase (right, with coloured overlay).

In order to link the results of the revealing of the γ and γ’ phases in the studied material with the processing of their microstructural images, selected examples of the best results have been presented in Figure 3. The images on the left (grey-scale, initial images) of Figure 3 were found to be the most appropriate for automated segmentation because they most clearly delineate the γ’ phase particle boundaries. The image preprocessing and then segmentation have been conducted with the computer-aided image analysis program Met-Ilo [25]. The microareas of the sample of the CMSX-4, selectively etched to remove γ’ phase, can be seen in Figure 3 (on the left). The required segmentation of the γ’ phase (Figure 3, on the right, images with coloured overlay) has been obtained after application of the following image transformations: a median filter (5 × 5 pixels), then a normalization of histogram and later a maximum entropy. Finally, a small manual touch-up was employed.

## 4. Discussion

Many preparation method deficiencies have arisen because the preparation methods have been developed empirically. Even having great metallographic samples and knowing the composition of the solution for etching from the literature, you can obtain unsatisfactory results when observing the etched surface of the sample because of lack of skill and experience during the etching and observation of the microstructure. One needs to know, for example, if the reagent should be prepared as fresh one; if the solution for etching is ready (all the components are enough dissolved); if the components for the etchant preparation are strong and, as a result, the time of etching should be short; if the specimen has been well dried after the etching, as otherwise, one will observe undefined objects on the surface of the sample; if one requires to lightly etch the specimen once again before the observation by means of SEM; and/or if one has applied the best combination of possible conditions for imaging. 

Far too little attention has been paid to the metallographic preparation and etching as important components in a system of repeatable and reproducible material investigations. 

Measurements in metallography can present a complex challenge. The condition of the item being measured (the polished and etched specimen), as well as the equipment used to perform the measurement can be relatively uncontrolled and each factor can add to uncertainty. Even image processing algorithms, and often automatic thresholding tools, can vary greatly and affect the measurement results. Image processing could be a time-consuming task if you do not devote enough time for sample preparation and good revealing of the interesting phases/ particles/ areas in the microstructure. 

Electrolytic extraction has been used by several scientists, e.g., [28,29,30], to determine the total weight fraction of the γ’ with reported success. The presented upshots of various etchings in the case of the CMSX-4 superalloy showed that the quite good results of the γ’ phase determination could be also achieved by means of the three chemical etching methods (No. 2, 6 and 9 in Table 2, Figure 3). 

The CCD-generated electronic image captured with the microscope results in a dramatic increase in the ability to enhance features, extract information or modify the image. The accuracy of quantitative metallography depends not only on the size and area measurement method but also on the selected etchant and etching conditions. Working out satisfactory and reproducible etching procedures requires time and patience. The main purpose of this article is to illustrate how different etchants and etching conditions can lead to different results in microstructural observation of the phases and microstructural constituents present in the nickel-based superalloy CMSX-4. The authors recommend especially the application of etchings no. 2, 6 and 9 for quantitative metallography with the use of a computer-aided image analysis program, for the reason that their application provides the best results in realistic measurements of the γ’ particle size distribution in an almost automatic manner with a little hand-made correction (Figure 3). 

The application of the other reagents (except for no. 2, 6 and 9, Table 2) for the γ’ phase quantitative evaluation may provide more difficulties in their image analysis, i.e., too much complicated and even impossible semi-automatic segmentation of the γ’ phase. Additionally, the results of the volume fraction of the γ’ phase based on measurements of the microstructures obtained with the etchants dissolving the γ matrix (no. 4, 7, 8, 11, 12, 13) will be overestimated.

## 5. Conclusions

According to the observations made during the performed activities to reveal the microstructure of the CMSX-4 superalloy, the following best practices for achieving the microstructural results of a Ni-based superalloy have been established:

- There are difficulties in the ascertaining of the real and clear microstructure of the superalloy (especially for SEM observation), whence some skills and experience are required;
- Most of the applied etchants provided good results in optical microscopy observation, but only few contribute in the ascertaining of the real and clear microstructure of the superalloy for SEM observation;
- The effectiveness of a reagent for etching should be verified experimentally using various conditions of etching and times of etching;
- It is possible to find a reagent which is recommended for a Ni-based superalloy, but in reality, it interacts with the surface of the material too aggressively (etchants no. 8, Table 2); it is usually a good idea to start with the weakest solution;
- In the case of a mounted specimen separation between the specimen and the mounting compound, the result can be ‘bleeding’ of the residual etchant or water and subsequent staining;
- To avoid the presence of unexpected objects on the surface of the sample during observation and imaging, try to rinse the sample after etching carefully;
- If additional etching time is required (especially for SEM observation), start with 1/2 second etching;
- The sample free of scratches and any kind of embedded contaminants has contributed to the increase in the effectiveness of the etching process, as well as the observation and imaging of the microstructure;
- Comparing the results of the application of the 14 reagents, it can be seen (Figure 2) that well visible phases and microstructural constituents in the whole field of view with the use of SEM have been provided by the application of etchants no. 2, 6 and 9.

On the basis of the performed preprocessing and segmentation of the γ’ phase precipitates (Figure 3), the following observations have been made:

- The accurate and efficient quantitative analysis of the microstructure in superalloys by means of image analysis is a challenge;
- The etching, as well as ascertaining the real and clear microstructure of the superalloy, has played a vital role in bringing about correct and uncomplicated separation of the features of interest (the γ’ phase precipitates) in an image from the background;
- Preprocessing of the image of the microstructure of the superalloy and segmentation of the γ’ phase precipitates have been associated with difficulties because, in many cases, the etchant used to highlight the γ or the γ’ phase can etch the material at different rates within the metallographic sample, varying the contrast in intensity for the γ and γ’;
- The presented examples of a proposal of the γ’ phase precipitates segmentation (Figure 3) are a good illustration of the ability to enhance features, as well as extract information from the digital image of the microstructure;
- Image segmentation performed by hand in the case of γ’ phase precipitates would be time- and work-consuming, making its quantitative characterization very expensive. That is why the search for semi-automated or automated and simultaneously precise segmentation of the γ’ phase precipitates is so important;
- There is a similarity between the attitudes expressed by a quantitative determination of γ’ phase using etchants dissolving the γ’ phase in this study (Figure 3) and those described in the works [15,16].

The findings of this study suggest that, with the knowledge of the material and experimenting with different preparation options as well as observations of the microstructure, we would be able to correctly reveal the superalloy microstructure, carry out an accurate measurement of the feature of interest using image analysis methods and finally interpret the obtained results. Further research in this field would be of great help to all professions related to Ni-based superalloy manufacturing, processing and applications.

## Figures and Tables

**Table 1:** Materials and parameters for specimen preparation.

Stages	Surface	Abrasive/Size	Load (N)/Specimen	Base Speed (rpm)/Direction	Time (Min)
Stage 1	Carbimet disc	P180 grit SiC, water cooled	25	350/complementary direction (platen and specimen holder both rotate in the same direction)	Until plane surface
Stage 2	Carbimet disc	P320 grit SiC, water cooled	25	350/complementary direction	1/2
Stage 3	Carbimet disc	P600 grit SiC, water cooled	25	350/complementary direction	1/2
Stage 4	Ultra-Pol cloth	9-μm Metadi Supreme–diamond suspension	25	150/opposite direction (platen and specimen holder rotate in opposite direction)	4
Stage 5	Trident cloth	1-μm Metadi Supreme–diamond suspension	20	150/opposite direction	4
Stage 6	Chemomet pads	Masterprep 0.05-μm alumina suspension	20	150/opposite direction	1
Stage 7	Microcloth	Masterprep 0.05-μm alumina suspension	-	Vibratory polishing	60

**Table 2:** Etchants applied for the Ni-based superalloy.

Etchant	Composition	Notes
1	45 gm FeCl_3_, 9 gm Cl_4_CuH_8_N_2_, 150 mL HCl, 75 mL distilled H_2_O	chemical/immerse
2	50 mL distilled H_2_O, 50 mL C_2_H_6_O, 50 mL HCl, 10 g CuSO_4_	chemical/immerse
3	5 mL H_2_SO_4_, 3 mL HNO_3_, 90 mL HCl	chemical/immerse
4	10 mL H_3_PO_4_, 50 mL H_2_SO_4_, 40 mL HNO_3_	electrolytic
5	5 mL H_2_SO_4_, 8g CrO_3_, 85ml H_3_PO_4_	electrolytic
6	20 mL HNO_3_, 60 mL HCl	chemical/immerse
7	5 g FeCl, 2 mL HCl, 100 mL C_2_H_6_O 95%, 100 mL CH_3_OH 95%	chemical/immerse
8	10 g C_2_H_2_O_4_, 100 mL distilled H_2_O	electrolytic
9	10 mL HNO_3_, 50 mL HCl, 60 mL glycerine	chemical/immerse
10	50 mL HCl, 50 mL C_2_H_6_O 95%, 50 mL CH_3_OH 95%	chemical/immerse
11	1 g CH_4_N_2_S, 1 mL H_3_PO_4_, 1 l distilled H_2_O	electrolytic
12	2/10 g CrO_3_, 100 mL distilled H_2_O	electrolytic
13	A) 2 g CrO_3_, 100 mL distilled water	electrolytic chemical/immerse
14	B) 4 g NaOH, 10 g KMnO_4_, 85 mL distilled water 100 mL HCl, 0.5 mL H_2_O_2_ (30%)	chemical/immerse
